# *Candida* spp. Determination and Th1/Th2 Mixed Cytokine Profile in Oral Samples From HIV+ Patients With Chronic Periodontitis

**DOI:** 10.3389/fimmu.2019.01465

**Published:** 2019-06-27

**Authors:** Sarah M. Lomeli-Martinez, Eulogio Valentin-Goméz, Juan J. Varela-Hernández, Monserrat Alvarez-Zavala, Karina Sanchez-Reyes, Moises Ramos-Solano, Rodolfo I. Cabrera-Silva, Victor M. Ramirez-Anguiano, Manuel A. Lomeli-Martinez, Silvia Y. Martinez-Salazar, Luz A. González-Hernández, Jaime F. Andrade-Villanueva

**Affiliations:** ^1^Department of Wellbeing and Sustainable Development, Centro Universitario del Norte, University of Guadalajara, Colotlán, Mexico; ^2^Biological and Agricultural Sciences Ph.D. Program, Centro Universitario de la Ciénega, University of Guadalajara, Ocotlán, Mexico; ^3^GMCA Research Unit, Department of Microbiology and Ecology, University of Valencia, Valencia, Spain; ^4^Severe Infection Group, Health Research Institute “La Fe,”, Valencia, Spain; ^5^Department of Medical and Life Sciences, Centro Universitario de la Ciénega, University of Guadalajara, Ocotlán, Mexico; ^6^HIV and Immunodeficiencies Research Institute, Clinical Medicine Department, Centro Universitario de Ciencias de la Salud-University of Guadalajara, Guadalajara, Mexico; ^7^Department of Integrated Dentistry Clinics, Centro Universitario de Ciencias de la Salud, University of Guadalajara, Guadalajara, Mexico; ^8^HIV Unit Department, University Hospital “Fray Antonio Alcalde,” University of Guadalajara, Guadalajara, Mexico

**Keywords:** *Candida* spp, chronic periodontitis, HIV, HAART, cytokines

## Abstract

**Background:** Chronic periodontitis (CP), caused by bacteria and fungi, appears in up to 66% of HIV-patients. The impact and association of HIV-treatment (HAART) and *Candida* itself has not been properly evaluated in the development and progression of CP. The immunopathogenesis is characterized by CD4^+^ T-cells activation and the balance between the T-helper 1 (Th1) and T-helper 2 (Th2) or a mixed cytokine profile. Currently, the associated causes of an immune response in HIV-patients with CP is controversial. Our aims were the determination of *Candida* spp. and cytokine profile in oral samples from HIV-positive patients with CP, considering the CD4^+^ T cells levels and HAART use.

**Methods:** From 500 HIV-positive patients evaluated, 228 patients were enrolled. Patients were separated in groups: (A) *n* = 53 (≤200 CD4^+^ T-cells on HAART); (B) *n* = 57 (≤200 CD4^+^ T-cells without HAART); (C) *n* = 50 (>200 CD4^+^ T-cells without HAART); (D) *n* = 68 (>200 CD4^+^ T-cells on HAART). *Candida* spp. were isolated from the oral biofilm and crevicular fluid in CHROMagar and confirmed by endpoint PCR. Cytokine levels were measured by beads-based immunoassay in saliva by flow cytometry.

**Results:** 147 patients (64.5%) were positive to *Candida spp*. and 204 strains were isolated; 138 (67.6%) were *C. albicans* and the remaining *C. non-albicans* species (*C. glabrata*>*C. tropicalis*>*C. krusei*>*C. dubliniensis)*. In this study, CHROMagar showed good sensitivity (95%) but poor specificity (68%); since of the 152 samples identified as *C. albicans*, only 131 were confirmed by PCR; from the 10 samples identified as *C. glabrata*, only six were confirmed. Finally, of the 42 samples detected as *C. tropicalis*, only five were confirmed. When evaluating *Candida* spp. presence, group A and D had higher isolation, while group B had the highest species diversity. Whereas, group C had a significant reduction of *Candida* spp. Despite the presence of *Candida* and HAART, we found a Th1/Th2 hybrid profile in the saliva of patients with low CD4^+^ T-cell count (group A).

**Conclusion:** Abundance and diversity of the *Candida* spp. detected in HIV-patients with CP could be related to HAART and low CD4^+^ T-cells levels. Also, the immunosuppression might promote a local Th1/Th2 hybrid cytokine profile.

## Introduction

In HIV positive (HIV+) patients, periodontitis represents one of the first opportunistic pathologies that manifest in the oral cavity, appearing in up to 66% of these patients (1). Periodontitis is a disease caused by both Gram-positive and negative bacteria and oral fungi. It is characterized by inflammation in the gum and adjacent tissues, which causes the destruction of teeth structural supports (2, 3). The mechanisms of damage include both the direct tissue damage caused by the bacterial products of the dental biofilm and the indirect damage produced by the bacterial induction of host inflammatory and immune response (2–5). The immunopathogenesis of periodontitis is orchestrated by the innate and adaptive immune response, mainly through the activation of CD4^+^ T cells and the balance between the T helper 1 (Th1) and T helper 2 (Th2) subtypes or a mixed cytokine profile (5, 6). Th1 signature cytokines are interleukin-2 (IL-2) and Interferon-gamma (IFN-γ), while Th2 cells secrete their signature cytokine interleukin 4 (IL-4) plus interleukin 5 (IL-5) and interleukin 10 (IL-10). It is well-known, that oral response against fungi in HIV negative (HIV-) subjects is orchestrated by a Th17 profile, this cytokine profile promote inflammation through the induction of a Th1 cytokine profile (inflammatory), recruitment of neutrophils, and production of reactive oxygen species such as nitrogen oxide (NO). This oral microenvironment is produced by infiltrating immune cells that are stimulated by the presence of opportunistic microorganisms and commensal bacteria that are part of the subgingival biofilm. This proinflammatory microenvironment (IL-1β, IFN- γ, IL-6, NO) is associated to bone loss, and the induction of a Th2 profile is a compensatory mechanism that controls the inflammation and promotes immune homeostasis (7).

Since HIV infection is characterized by the depletion of CD4^+^ T cells and several changes in the whole cellular and humoral immune response that could lead to an immunodeficient state, which may allow subgingival colonization by different pathogens, HIV+ patients exhibit more oral manifestations that HIV- subjects (8). One of such pathogens is *Candida*, which can aggregate jointly with other bacteria to the subgingival biofilm and attach to the epithelial cells of patients with periodontal disease (8–10). *C. albicans* is the most frequent species identified in patients with periodontal disease. However, other species have also been found like *C. tropicalis, C. glabrata, C. krusei*, and *C. guilliermondii* (8, 10, 11). Also, it has been suggested that *Candida* spp. play a role in both the pathogenesis and the severity of periodontal disease (11–13). These last aspects gather importance in HIV+ patients. Both the local and systemic effects of the periodontal disease and the chronic immune activation associated with a co-infection are crucial factors in AIDS severity and progression. Furthermore, periodontal bacteria favor Epstein-Barr virus and Kaposi's sarcoma-associated herpesvirus reactivation (14).

It has been reported that CD4^+^ T cells count >200 cells/μL increase the probability to acquire oral manifestations such as oropharyngeal candidiasis (OPC) (15, 16). A possible explanation to this susceptibility is by the depletion of Th17 (particularly in the gut) and an increase of a Th2 profile in the mucosa of HIV+ patients, as both factors are associated with susceptibility to mucosal candidiasis (7, 17, 18). In OPC, CD8^+^ T cells are recruited to the mucosa in response to Candida infection, promoting a proinflammatory microenvironment characterized by a Th1 cytokine profile (19). This compensatory response in OPC is reflected in a Th1/Th2 cytokine profile, however, the establishment of a similar immune profile has not been described in CP.

In our knowledge, HIV+ patients with CP have been studied without considering *Candida* infection, nor the clinical conditions of the patients, such as CD4^+^ T cell count and HAART. In addition, they do not evaluate if CD4^+^ T cell count or HAART could modify the cytokine environment or promote atypical *Candida* spp. colonization in the mouth. Several studies analyzed the role of *Candida* spp. in mucosal infection in HIV+ patients, however, most of them are focused in OPC, while this comorbidity has been studied microbiologically and immunologically, other infections driven by *Candida* spp. such as CP, remain poorly known. We hypothesized that *Candida* spp. colonization in HIV+ patients with CP is associated with a Th1/Th2 profile independently of the immune state and the presence of HAART, however, these two last factors are related to the diversity of *Candida* spp.

Our aims were the determination of *Candida* spp. and cytokine profile in oral samples from HIV+ patients with CP, considering the CD4^+^ T cells levels and HAART use.

## Materials and Methods

### Study Population

We performed a cross-sectional (observational) study including 500 HIV+ patients with untreated CP in the last 3 months. HIV patients were recruited from the HIV clinic of the tertiary care university hospital “Antiguo Hospital Civil de Guadalajara—Fray Antonio Alcalde” (a 1,000-bed teaching hospital in Western Mexico) from February to September 2017. Non-inclusion criteria were pregnancy, breastfeeding, no sign of CP, oral candidiasis, and use of dental prostheses. This study was carried out in accordance with the recommendations of the Declaration of Helsinki, World Medical Association with written informed consent from all subjects. The protocol was approved by the ethics committee in research of the “Antiguo Hospital Civil de Guadalajara—Fray Antonio Alcalde” with registry number 020/16 (20). All subjects gave written informed consent in accordance with the Declaration of Helsinki.

The sample size was calculated using a finite population proportion formula based on the prevalence previously reported (21). Detailed sociodemographic, immunological, and health-related information were obtained during the scheduled medical visit.

Selected patients were grouped as follows: Group (A) patients with CD4^+^ ≤200 cells/μL on HAART; Group (B) patients with CD4^+^ ≤200 cells/μL without HAART; Group (C) patients with CD4^+^ >200 cells/μL without HAART; and Group (D) patients with CD4^+^ >200 cells/μL on HAART.

### Periodontal Evaluation

The periodontal evaluation was performed by a periodontist with a North Carolina periodontal probe number 12 (Hu-Friedy). This analysis evaluated the mesiobuccal, buccal, distobuccal, mesiolingual, lingual, and distolingual site of each tooth (except the third molars). In each site: sounding depth, Clinical Attachment Level (CAL) and bleeding on probing were evaluated. Periodontitis diagnosis was established according to the Disease Control/American Association of Periodontology Classification (22), which considers the CAL scale.

### Sample Collection

Crevicular fluid and subgingival biofilm were obtained from the most severe site in each oral quadrant at the base of the periodontal pocket. The crevicular fluid was obtained by introducing filter paper for 60 s. Subgingival biofilm was obtained with a sterile Gracey curette (Hu-Friedy). For *Candida* spp. identification, crevicular fluid, and subgingival biofilm were collected in thioglycolate medium (BD) tubes. Non-stimulated saliva samples were collected in a clean 2 mL amber tubes and stored at −70°C. All samples were collected before noon after periodontal evaluation.

### *Candida* spp. Detection by CHROMagar®

Crevicular fluid and subgingival biofilm were streak-plated in CHROMagar Candida® medium, incubated at 36°C for 48 h. Identification was carried out by colorimetric changes as specified by the manufacturer's instructions. All CHROMagar were analyzed by a certified microbiologist.

### Endpoint PCR From Yeast Colonies

Endpoint PCR was performed in yeast colonies as previously described (https://openwetware.org/wiki/Smolke:Protocols/Yeast_Colony_PCR). Briefly, primary *Candida* spp. acquired from the CHROMagar were re-cultured overnight in yeast 1% extract, 2% peptone, and 2% dextrose (YPD) medium. Yeast colonies were harvested with a sterile pipette tip and resuspended in 3 μL of freshly made NaOH (20 mM). Cells were lysed and incubated at 95°C for 10 min. The 3 μL were used as molds for the PCR reaction, which was performed in a total volume of 25 μL. PCR mix was made with 5 mM dNTPs, 15 mM MgCl_2_, and Taq polymerase following the manufacturer's instruction (MyTaq^TM^ DNA Polymerase Kit, Bioline). Three percent of DMSO was added to improve reaction as previously described (23) and 10 mM of primers specific for every species (Table 1).

**Table 1:** Primer sets used for *Candida* spp. PCR detection.

***Candida*spp**.	**Primer set**	**References**
*C. albicans*	INT1 5′ AAGTATTTGGGAGAAGGGAAAGGG 3′	(24)
	INT2 5′ AAAATGGGCATTAAGGAAAAGAGC 3′	
*C. tropicalis*	CTf 5′ TGATAGTTAGGAAAGATCAGGTG 3′	(24)
	CTr 5′ CACACACATGGGATATGTT 3′	
*C. glabrata*	CGf 5′ ACATATGTTTGCTGAAAAGGC 3′	(24)
	CGr 5′ AGAAGTCCTGAACACTAAGAAAAAGT 3′	
*C. parapsilosis*	CPf 5′ AGGGATTGCCAATATGCCCA 3′	(24)
	CPr 5′ GTGACATTGTGTAGATCCTTGG 3′	

Endpoint PCR was performed in a MiniCycler MJ Research thermal cycler. PCR program was as follows: 5 min of denaturalization at 95°C, 30 cycles of 30 s at 94°C (denaturalization), 30 s at 50°C (hybridization), and 30 s at 72°C (elongation). Afterward, a final elongation at 72°C for 10 min was performed (24).

### Electrophoresis

DNA fragments from the PCR reaction were separated in 2% agarose gel (CONDA Micro and Molecular Biology®) prepared with TAE buffer (Tris-acetate 40 mM, pH 8.5; EDTA 0.05 mM). Ten microliter of Red Safe® (iNtRON) were added. Photographs were taken with a UVITEC transilluminator.

### Cytokine Determination

Saliva samples were centrifuged at 1,000 g for 10 min, the supernatant was recollected and used for the assay. LEGENDplex Human T Helper Cytokine Th1/Th2 Panel kit (BioLegend) was used for cytokines detection. The evaluated cytokines were: IL-2, IL-4, IL-5, IL-6, IL-10, IL-13, IFN-γ, and Tumor Necrosis Factor-alpha (TNF-α). The assay was performed following manufacturer recommendations. Samples were read using the NxT Attune flow cytometer (Applied Bioscience) and analyzed with the LEGENDplex v8 Software (BioLegend).

### Statistical Analysis

Data were analyzed using Statistical Package for the Social Scientist (SPSS) version 23 (IBM) and GraphPad Prism 6 software. Medians and interquartile ranges (IQR) or means and standard deviations (SD) were reported depending on data distribution. Sociodemographic and *Candida* spp. presence data were compared with either Student's *t*-test, Fisher exact, or Chi-square, according to the variable analyzed; binary linear regression analysis was performed to estimate the association of candida presence, considering the following variables: use of HAART, age, gender, level of CD4^+^ T cells (> or ≤200 cells/μL), tobacco, alcohol, and drug use; and Cohen's kappa coefficient (κ) was reported. The cytokines differences between the different groups were estimated using the Kruskal-Wallis test with Dunn's Multiple Comparison test. Shapiro-Wilks normality test was significant, so the data distribution was considered non-Gaussian. *p*-values < 0.05 were considered significant.

## Results

### Group Study Characteristics

**Figure 1:**
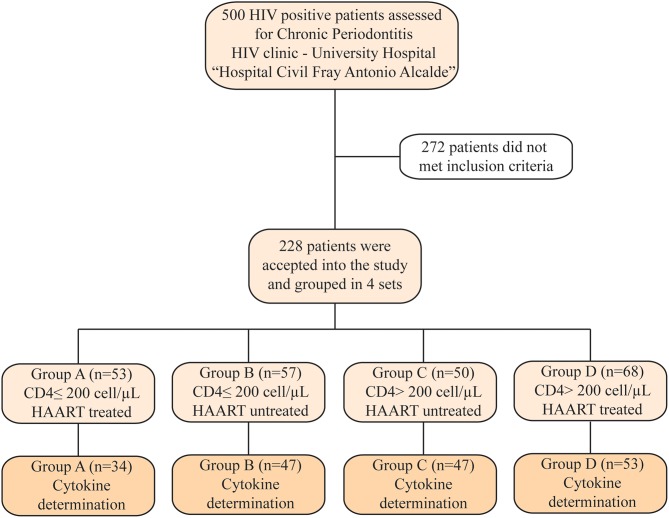
Flowchart of patient recruitment and distribution. Two hundred twenty-eight patients were selected after exclusion criteria and separated into four groups. Due to low quality in saliva samples, we were not able to determine the cytokines profile in the samples from all patients included in this study.

Periodontal evaluation was performed in 500 HIV+ volunteer patients. After assessing the selection criteria, 228 patients were included in the study. Next, patients were divided into different groups: 53 patients were assigned to Group A, 57 patients to Group B, 50 patients to Group C, and 68 patients to Group D (Figure 1).

Cytokine determination in saliva was performed only in 34 from Group A, 47 from Group B, 47 from Group C, and 53 from Group D; this due to lack of enough sample or samples that were blood-stained. It was observed a significant difference among age, CD4^+^ T cells count, CD4/CD8 ratio, viral load, duration of HAART, and grades of severity according to the CAL scale. Regarding age, the group with the older patients was group D (43 ± 11 years; *p* = 0.05). Patients with ≤200 CD4^+^ T cells had a median of 17 months under HAART compared to the 38 months among patients with >200 CD4^+^ T cells (*p* = 0.0001). Furthermore, this last group also had an undetectable viral load (*p* = 0.0001). In addition, group B—with ≤200 CD4 ^+^ T cells- presented less “mild” cases and more “severe” cases, according to the CAL classification, when compared against group C—with >200 CD4^+^ T cells-, both groups without HAART (*p* = 0.05).

In respect to gender, the majority of our patients were men (4:1). Because in group B there was only one woman, a significant difference was found but this statistic finding should be considered with caution. We did not find differences among the four groups in terms of the habits of smoking, drinking or drug use, but is important to emphasize that more than 62% of patients used tobacco, that is already considered as a risk factor for CP. Besides, diabetes was absent in more than 93% in all groups, that is relevant considering that it is another known risk factor for CP and was absent in the majority of our patients (25). The treatment scheme in the groups under HAART was based mainly in non-nucleoside reverse transcriptase inhibitors (NNRT), followed by protease inhibitors (PI), and integrase inhibitors; nonetheless, there were no differences statistically significant between the groups. Complete sociodemographic data is described in Table 2.

**Table 2:** Sociodemographic characteristics including all patients.

	**Group A**	**Group B**	**Group C**	**Group D**	*p*-value^*^
	***n* = 53**	***n* = 57**	***n* = 50**	***n* = 68**	
Age (years)	37.3 ± 9	36.9 ± 8	37 ± 12	42.6 ± 11	0.05
Mean (SD)					
Gender					0.05^#^
Female	10 (19%)	1 (2%)	11 (22%)	15 (22%)	
Male	43 (81%)	56 (98%)	39 (78%)	53 (78%)	
Tobacco use:					ns
Yes	34 (64%)	38 (67%)	31 (62%)	47 (69%)	
No	19 (36%)	19 (33%)	19 (38%)	21 (31%)	
Drugs use:					ns
Yes	34 (64%)	31 (54%)	24 (48%)	41 (60%)	
No	19 (36%)	26 (46%)	26 (52%)	27 (40%)	
Alcohol use:					ns
Yes	40 (76%)	41 (72%)	38 (76%)	53 (78%)	
No	13 (24%)	16 (28%)	12 (24%)	15 (22%)	
Diabetes:					ns
Present	2 (4%)	4 (7%)	2 (4%)	4 (6%)	
Absent	51 (96%)	53 (93%)	48 (96%)	64 (94%)	
CD4 ^+^ (cells/mL)	41	38	366	503	0.0001
Median (IQR)	(11–109)	(19–89)	(264–552)	(354–697)	
CD4/CD8 ratio	0.1	0.11	0.42	0.54	0.0001
Median (IQR)	(0.05–0.17)	(0.05–0.16)	(0.25–0.67)	(0.40–0.77)	
Viral load (copies/mL)	31,400	149,000	30,600	38	0.0001
Median (IQR)	(428–297,250)	(57,800–411,436)	(183–77,750)	(20–78)	
Type of HAART:					ns
No medication	–	100%	100%	–	
PI-based	19 (36%)	–	–	23 (34%)	
NNRT-based	18 (34%)	–	–	33 (48%)	
INI-based	7 (13%)	–	–	4 (6%)	
Other	9 (17%)	–	–	8 (12%)	
Duration in treatment (months)	17 (6–23)	–	–	38 (24–104)	0.0001
CAL classification:					
Mild	7 (13.2%)	7 (12.2%)	13 (26%)	12 (17.6%)	0.05
Moderate	15 (28.3%)	14 (24.5%)	17 (34%)	24 (35.2%)	ns
Severe	31 (58.4%)	36 (63.1%)	20 (40%)	32 (47%)	0.05

* *Fisher's exact test, Chi-squared test, ANOVA, Kruskal-Wallis, and Mann-Whitney U-tests*.

# *Statistical significance was found only with Group B because it only had one female patient; however, between the rest of the groups, there were no statistical differences; SD, standard deviation; IQR, interquartile range; ns, no significance. Group A: ≤ 200 CD4^+^ on HAART; group B: ≤200 CD4^+^ without HAART; group C: >200 CD4^+^ without HAART; group D: >200 CD4^+^ on HAART*.

After the logistic regression analysis, we found a significantly association with the presence of *Candida* spp. with levels ≤200 cells/μL of CD4^+^ T cells (OR: 3.59 CI 95% 1.89–6.80; *p*=0.0001), the use of PIs (OR: 2.67 CI 95% 1.13–6.29; *p* = 0.025) and NNTRIs (OR: 2.99 CI 95% 1.37–6.49; *p* = 0.006; κ = 0.408).

### *Candida* spp. Varies Between Groups and Identification Methods

HIV infection is associated with an increased colonization rate by *Candida* spp. as well as the development of other diseases, such as periodontal disease. Our first goal was to identify the *Candida* spp. present in the different groups of HIV+ patients with periodontal disease.

From the 228 patients included in the study, 147 (64.5%) were positive to *Candida* spp. In total 204 strains were isolated, of which 138 (67.6%) were *C. albicans*, and the remaining 66 (32.4%) had *C. non-albicans* species. The identified *non-albicans* species were: 48 (23.6%) *C. glabrata*, 10 (4.9%) *C. tropicalis*, 7 (3.4%) *C. krusei*, and 1 (0.5%) *C. dubliniensis*.

In 145 (71.1%) of the total isolated strains, only one type of *Candida* was isolated, while the remaining 59 isolated strains (28.9%) more than one type of *Candida* were isolated. The most common combinations were: *C. albicans* + *C. glabrata* (19.6%), *C. albicans* + *C. krusei (*4.9%), *C. glabrata* + *C. tropicalis* (2%), and *C. albicans* + *C. glabrata* + *C. tropicalis* (1.5%). When comparing all combinations between the study groups, Group C had significantly fewer combinations in comparison with Group D (*p* = 0.011). The distribution of *Candida* spp. strains separated by groups are shown in the Supplementary Table 1.

#### Identification of Candida Species With Different Methods

When carrying out this study, it was necessary to use different methods of identification for *Candida* spp., including conventional methods as well as molecular techniques. At the beginning, with streak-plating technique by CHROMagar, *C. albicans, C. glabrata*, and *C. tropicalis* were identified in 152, 10, and 42 samples; respectively. However, of the 152 *C. albicans* identified, molecular confirmation was positive in 131 of these samples. Therefore, PCR for the other *Candida* species was performed in the remaining 21 samples; 14 of these samples were positive for *C. glabrata*, and 3 for *C. tropicalis*. The last four samples were not identified by PCR. From the 10 samples identified as *C. glabrata*, six were confirmed by molecular techniques. The remaining four samples were tested by PCR for the other *Candida* species, two were identified as *C. albicans* and the others as *C. tropicalis*. The last 42 samples were detected as *C. tropicalis*, but only five were confirmed by PCR. In the other 37 samples, 28 were identified as *C. glabrata*, and five as *C. albicans*. Again, four samples were not identified to any *Candida* spp. by PCR.

**Figure 2:**
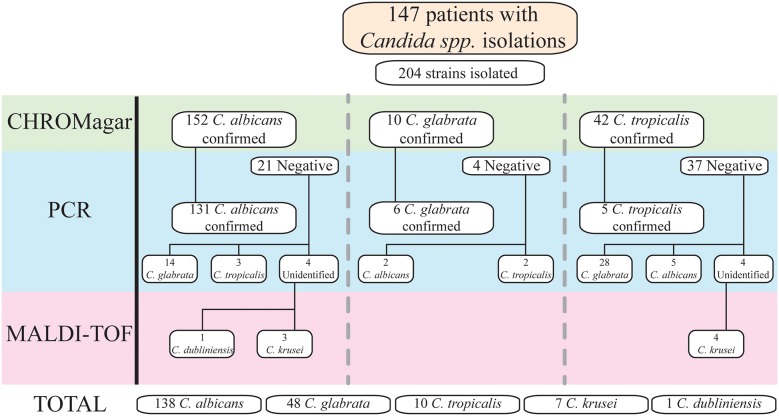
*Candida* spp. identification by methods. All samples were cultivated first in CHROMagar for initial identification. Afterward, all identified *Candida* spp. by traditional microbiologic methods were evaluated by PCR technique to confirm the species. If the PCR for the specific species found in CHROMagar was negative, further PCRs were made for other *Candida* spp. Any *Candida* spp. that was not confirmed by PCR, due to lack of the primers specific for that species, were identified through MALDI-TOF technique.

The eight samples that were negative in all PCRs were sent to “La Fe” Hospital in Valencia, Spain. *Candida* spp. was detected through matrix-assisted laser desorption/ionization time-of-flight mass spectrometry (MALDI-TOF MS). From the four samples detected as *C. albicans* by streak-plating technique, one was identified as *C. dubliniensis* and the remaining as *C. krusei*. The four samples detected as *C. tropicalis* by plating were confirmed as *C. krusei* by MALDI-TOF MS. A summary is provided in Figure 2.

With this information, we compared the accuracy of CHROMagar Candida medium to identify *Candida* spp. with streak-plating technique against PCR; results are shown in Table 3.

**Table 3:** Capacity of *Candida* spp. identification with CHROMagar Candida Medium.

**Marker**	**Value**	**95% confidence interval**
Sensibility	95%	89.8–97.9%
Specificity	68%	55.6–79.1%
Positive likelihood ratio	2.98	2.09–4.26
Negative likelihood ratio	0.07	0.04–0.16
Positive predictive value	86%	81.4–89.9%
Negative predictive value	87%	75.4–93.1%
Accuracy	86%	80.8–90.7%

#### *Candida* spp. and Colonization Varies Depending on CD4^+^ T Cells Count and HAART

In the course of HIV infection, the rate of Candida infection is inversely related to the CD4^+^ T cells count. When evaluating *Candida* spp. between the groups, *C. albicans* and *C. glabrata* were identified in all of them; *C. krusei* was detected in Groups A, B, and D; *C. tropicalis* in Group B and D, while *C. dubliniensis* was present exclusively in Group B.

When analyzing the number of patients with *Candida* in each group, we found a significant reduction of *Candida* spp. in the Group C in comparison with the other groups (*p* ≤ 0.001 vs. A, B, D). As well, *C. albicans* was significantly diminished in Group C (*p* < 0.01 vs. A and B; *p* < 0.05 vs. D). For patients with *C. glabrata*, Group C had also a significant decrease in comparison with B and D (*p* < 0.05 and *p* < 0.01, respectively). Data are shown in Table 4.

**Table 4:** Patients with presence of *Candida* spp. in each group.

	**Group A**	**Group B**	**Group C**	**Group D**	***p*-value**
	***n* = 53 (%)**	***n* = 57 (%)**	***n* = 50 (%)**	***n* = 68 (%)**	
***Candida*** **spp**.	**36 (68)**	**45 (78)**	**13 (26)**	**51 (75)**	A vs. C^a^^***^
					B vs. C^a^^***^
					D vs. C^a^^***^
*C. albicans*	26 (49)	27 (47)	11 (22)	29 (43)	A vs. C^a^^**^
					B vs. C^a^^**^
					D vs. C^a^^*^
*C. glabrata*	9 (17)	11 (19)	2 (4)	15 (22)	B vs. C^b^^*^
					D vs. C^b^^**^
*C. tropicalis*	0 (0)	4 (7)	0 (0)	5 (7)	ns
*C. krusei*	1 (2)	2 (3)	0 (0)	2 (3)	ns
*C. dubliniensis*	0 (0)	1 (2)	0 (0)	0 (0)	n/a

*Groups were compared between each other for overall Candida infection and for each individual Candida spp. detected. Total Candida identification is shown with their percentage (round up) for every group. Statistically significant p-values are shown. For C. dubliniensis, the statistical comparison was not performed because only one group had this species (n/a). As not every patient in each group had Candida, the amount of Candida spp. detected and total patient per group differs*.

a Chi-squared test;

b Fisher's exact test;

* p < 0.05;

** p < 0.01;

*** *p < 0.001; ns, no statistical significance*.

### Th1/Th2 Mixed Cytokines Profile in the Saliva Is Mainly Promoted by Low CD4^+^ T Cell Count

Saliva samples from HIV+ patients with CP and *Candida* spp. growth were analyzed to evaluate if CD4^+^ T cell count or HAART modifies the local Th1 and Th2 related cytokine expression. With respect to Th1-related cytokines, IFN-γ was significantly higher in group A and B in relation to group D; the levels of TNF-α in group A were significantly higher against group D, and significantly higher to group B for IL-2. Finally, group B had significantly more IL-6 in comparison with group C and D.

Regarding Th2 signature cytokines, group A had a significant increase of IL-5 in comparison with group B and D; whilst both groups with low CD4^+^ T cell count (A and B) had a significant increase of IL-10 and IL-4 respect to group D. Group A was also significant with group C for IL-4. When comparing cytokine expression in each group, there was no difference for IL-13 (Table 5; Figure S1). We were able to observe a mixed increase of some Th1 and Th2 cytokines, although a major predisposition for a Th2 profile was found. Interestingly, the groups with more levels of cytokines overall are the ones with low CD4^+^ T cell count.

**Table 5:** Cytokine levels in saliva samples from HIV+ patients per group.

**Cytokine median ± IQR (pg/mL)**	**Group A**	**Group B**	**Group C**	**Group D**	***p-value***
**Th1 PROFILE**					
IFN-γ	11.79 (7.10–25.21)	7.89 (6.63–23.39)	7.07 (6.0–17.23)	7.07 (3.31–8.23)	A vs. D^***^
					B vs. D^*^
TNF-α	5.35 (5.05–9.54)	5.05 (4.03–8.77)	5.05 (4.57–7.54)	5.05 (1.98–5.25)	A vs. D^**^
IL-2	15.18 (15.18–19.65)	15.18 (4.84–15.18)	15.18 (4.84–16.47)	15.18 (4.84–16.32)	A vs. B^*^
IL-6	19.03 (4.26–59.96)	40.79 (5.09–145)	4.83 (2.43–28.91)	4.82 (3.37–5.37)	B vs. D^***^
					B vs. C^**^
**Th2 PROFILE**					
IL-4	15.65 (5.79–33.63)	9.9 (5.17–36.85)	5.75 (2.27–13.75)	5.17 (2.13–9.89)	A vs. D^**^
					A vs. C^*^
					B vs. D^**^
IL-5	4.08 (4.08–10.39)	4.08 (2.98–4.08)	4.08 (2.98–4.08)	4.08 (2.98–4.08)	A vs. D^**^
					A vs. B^*^
IL-10	2.89 (2.83–11.22)	4.17 (2.83–13.53)	2.83 (2.39–4.25)	2.83 (2.27–2.87)	A vs. D^*^
					B vs. D^**^
IL-13	13.48 (12.47–17.81)	12.47 (5.49–17.81)	13.08 (5.45–23.68)	8.43 (3.81–15.16)	ns

Measurements of Th1 and Th2 profile's cytokines levels in all groups. All values are reported in pg/mL. Median and interquartile range (IQR) are shown. Kruskal-Wallis test was performed to evaluate differences. The p-values with statistical significance are reported as follows:

* p < 0.5;

** p < 0.01;

*** *p < 0.001; ns, no statistical significance*.

## Discussion

This study is one of the few studies that measure CP in different groups considering the CD4^+^ T cell count and HAART. Because other studies divided their groups differently to ours, we separate and regroup our data as required to better compare our results with the literature.

In this study, patients with CP and CD4^+^ T cells ≤200 cells/μL had a major prevalence of *Candida* infection (73.7%) than the one reported in previous studies (11–13, 26). In addition, the number of patients, in this study, colonized with *C. albicans* (40.8%), *C. tropicalis* (3.9%), and *C. krusei* (2.2%) is less than the ones found previously (11, 26). However, similar results to ours were found by another group (27). Furthermore, we found a higher amount of *C. glabrata* colonization (16.2%) in comparison with the literature (11, 26–28).

Regarding the distribution of *Candida* spp. isolation, *C. albicans* (67.7%), *C. glabrata* (23.5%), and *C. krusei* (3.4%) had a major distribution than the one reported (13, 29, 30). On the other hand, the distribution that we detected for *C. tropicalis* (4.9%) was lower than previous papers (13, 29, 30). Like previous studies, we identified *C. dubliniensis* in one of the patients (0.5%) (26, 29, 30). This is the first study in Mexico that identifies *Candida dubliniensis* in HIV+ patients with CP; it was previously reported in USA (31) and this could increase the relevance of screening this species in Mexican populations with CP.

In this study, we required to use several techniques for *Candida* spp. identification. Currently, several authors have considered the use of CHROMagar Candida medium as a diagnostic test directly from the oral cavity (27, 28, 32). Taking advantage that we used this technique, we evaluated the CHROMagar effectiveness as a diagnosis technique. Since CHROMagar is a presumptive test, we confirmed our results with endpoint PCR and found a sensibility of 95%, a specificity of 68%, and an accuracy of 86% for CHROMagar. These results differ from the studies that suggest a sensibility and a specificity of 99% (33, 34). These discrepancies could be explained by the different saliva sampling techniques used: isotopes method (28, 30, 35), mouthwash method (11), filter papers method (12, 36); or the identification technique performed: biochemistry tests (11, 28, 30), chromogenic medium (12, 28, 36) PCR (29) or MALDI-TOF MS (27). Therefore, an acquisition technique should be standardized when CHROMagar is used. Nonetheless, considering our results we suggest that CHROMagar Candida Medium requires a confirmatory technique, such as PCR, to ensure the results, especially for *Candida non-albicans species*, as recommended by other authors (24, 29, 32).

It is known, that the interaction of various *Candida* spp. with bacteria from the dental plaque promotes bacterial colonization and proliferation in the periodontal pockets. This contributes to the progression of periodontal disease (8). We isolated in 71.1% of the patients a single *Candida* spp.; and two or more *Candida* spp. in 28.9% of our patients. During the periodontal evaluation, the most severe patients were the ones with two or more *Candida* spp. (CAL ≥5 mm). Also, we detected an increase distribution of the *C. albicans* + *C. glabrata* coinfection (19.6%) in comparison with other studies (26, 29). Our results suggest that a transition to coinfection with multiple *Candida* spp. is a factor that facilitates CP and increases severity. Clark et al. (29), did a study with HIV+ Mexican patients without a periodontal evaluation and through a different acquisition technique (mouthwash) and they found in 94.1% of their patients a single *Candida* spp. and two or more species in 6.5% of them. Furthermore, the most frequent *Candida non-albicans* species that we found was *C. glabrata* (23.6%) in comparison with Clark et al. (29), which found *C. tropicalis*. In this sense, the differences found between our study and Clark et al. (29) study could be related to potential geographical environmental factors; it has already been proposed that environmental factors could have importance in the type of *Candida* spp. in oral colonization, factors that could be relevant when we compared our results with the literature (37).

The use of HAART and the immune reconstitution have been associated with a significant reduction of viral load and opportunistic infections, including oral manifestations like oral hairy leukoplakia, Kaposi's sarcoma and oral candidiasis (10, 21). However, it remains controversial the effect of HAART in incidence and severity of the atypical periodontal diseases, such as linear gingival erythema, necrotizing periodontitis and CP (10, 21, 38, 39); the latter, probably caused by the known effect of HAART over the salivary flow (hyposalivation, xerostomia, or dysgeusia), which modifies the oral microbiota and facilitates *Candida* spp. colonization (40, 41); yet other studies could not find a correlation between *Candida* colonization and HAART usage (29, 30, 42, 43). In this sense, we evaluated if the presence of HAART affected the amount of *Candida* colonization in CP. We detected that groups with HAART, especially with the use of PIs and NNRTIs, had a significant increase of *Candida* colonization. Interestingly, we also found an increase in diversity. Therefore, we suggest that a secondary effect of HAART is the promotion of oral dysbiosis.

Concerning CD4^+^ T cell count, the groups with CD4^+^ T cells count ≤200 cells/μL had a significant increase of *Candida* spp. colonization in comparison with the groups with CD4^+^ T cell count >200 cells/μL (OR: 3.59; *p* = 0.0001). This finding is consistent with the literature (11, 44). In summary, CD4^+^ T cell count in HIV+ patients play a major role in the subgingival colonization of *Candida* spp. in CP. It has been reported that during periodontal disease the expression of CD4^+^, CXCR5^+^, and CCR5^+^ is increased (45, 46); the latter has also been reported increased in smokers (47). This overexpression facilitates the entry of HIV virions through dendritic cells (DC) and macrophages, which are the prevalent cells in the oral mucosa; this could cause major cell death, local inflammation, and promotion for pathogen colonization like *Candida*. On average, 66% of HIV+ patients in our study are current smokers, it seems that this continues to be an important factor for the development of periodontal disease, but in our study, it was not shown that tobacco influenced *Candida* colonization.

Lastly, we assessed the local cytokine environment occurring in HIV+ patients with CP. For this, we analyzed saliva samples and found that the patients had a mixed cytokine profile. Similar results have been found previously in the literature, however, the cytokine diversity has varied within the studies (17, 18, 48, 49). This could be attributed to discrepancies in selection criteria within the studies and the period of the disease -acute or chronic-. In addition, we saw that the most increase of cytokines occurred in the groups with low CD4^+^ T cell count. A plausible theory for this finding is that cytokine production could be innate-mediated. DC and macrophages could be the main cells producing cytokine as they have been observed to remain prevalent in oral mucosa during periodontal disease (45, 46). Although, other types of cells could be involved in the cytokine environment, such as innate lymphoid cells (ILC) and natural killer (NK) cells. Li and Reeves (50) showed that ILC are increased and activated in oral mucosa during simian immunodeficiency viral infection, these cells can produce several cytokines and we consider that the innate immune system should be studied further in CP, as well as evaluating the role of ILC and NK cells in human's oral cavity. As we previously mentioned, HIV+ patients have a dominant Th2 cytokine environment, irrespective of periodontal disease status (18), since the immune response against *Candida* spp. requires a plethora of immune cells (mainly CD8^+^ T cells); the presence of a Th1 cytokine profile seems to be essential in the development of a resolutive immune response in CP. It is unknown, whether the Th2 cytokines present in the Th2/Th1 profile remains as a consequence in the oral immune microenvironment by the HIV infection, or if they are a secondary immune reactivation in order to establish a homeostatic mechanism. Something that we did observe is that the Th1/Th2 profile is present in all groups colonized with one or more *Candida* spp., despite their clinical, and demographic differences.

Though it has been reported that smoking promotes a Th1/Th2 cytokine profile in the saliva of HIV+ patients with OPC (51), in our study this factor did not have a major impact in the establishment of the mixed cytokine profile, since the majority of our study population presented a Th1/Th2 cytokine profile yet only 40% of them reported smoking habits. We considered that the shift in the local immune response from a Th2 to a Th1/Th2 profile is linked directly to the presence of *Candida* spp. (regardless of colonization), this data is consistent with the previous cytokine profile reported in OPC (17). In a non-HIV context, the progression of CP evolves from Th1/Th2 to Th1/Th17 profile (52, 53). Unfortunately, in our study we did not evaluate the Th17 profile. Whether these results are an early phase, which will further develop into a Th1/Th17 cytokine profile remains unknown and an interesting question to be answered in future studies.

**Figure 3:**
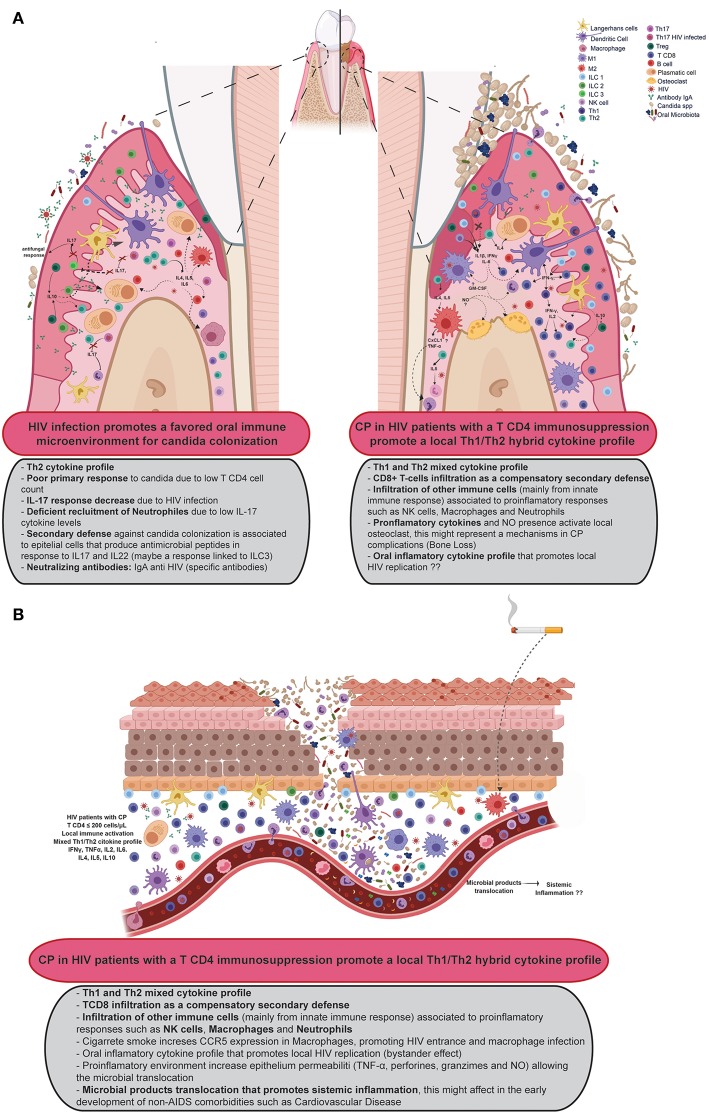
Proposal of the immune response in HIV patients with CP. **(A)** Suggests the progression to CP in the oral cavity while **(B)** shows a proposal for the involvement of CP in the systemic inflammation environment of HIV patients. Created with BioRender.

With these findings and what it has been reported in the literature (7), we proposed a potential diagram of the immune physiopathology of CP in patients with HIV (Figure 3). Briefly, the loss of Th17 cytokines and increased presence of the Th2 profile, facilitate the oral colonization with pathogens such as *Candida* spp. This colonization activates local innate immune cells -DC, macrophages, ILC, NK cells, and others- which secrete Th1 cytokines, promoting the presence of a Th1/Th2 profile, and also secreting chemoattractant (TNF-α, CXCL1, and GM-CSF), that allows neutrophils recruitment and increase their survivability (54). At the same time, the release of ROS elements promotes bone resorption by activated osteoclasts (55). This local immune behavior is strengthened by smoking habit that increases the CCR5 expression, which facilitates local HIV entrance to DC and macrophages, promoting an onset of a “trojan horse” effect and a major local inflammation. Altogether, this damage promotes the destruction of the teeth architecture and facilitates the translocation of microbial products that could be enhancing a systemic inflammation.

## Data Availability

All datasets generated for this study are included in the manuscript and/or the Supplementary Files.

## Ethics Statement

This study was carried out in accordance with the recommendations of the Declaration of Helsinki, World Medical Association with written informed consent from all subjects. The protocol was approved by the ethics committee in research of the Antiguo Hospital Civil de Guadalajara—Fray Antonio Alcalde with registry number 020/16. All subjects gave written informed consent in accordance with the Declaration of Helsinki.

## Author Contributions

SL-M performed the recruitment of patients, their periodontal evaluations, saliva acquisition, and original draft. SL-M, LG-H, VR-A, and ML-M helped with the diagnosis, evaluation, and assessment for inclusion or exclusion of patients for this paper. SL-M, EV-G, and JV-H performed PCRs and Candida identification. MA-Z, KS-R, MR-S, and RC-S performed cytokine determination and analysis. SL-M, JV-H, EV-G, RC-S, MA-Z, KS-R, MR-S, LG-H, and JA-V literature search, writing, review, and editing. SL-M, RC-S, MA-Z, KS-R, and MR-S developed the tables and figures for this manuscript. EV-G, SM-S, LG-H, and JA-V acquired the funds for this project.

### Conflict of Interest Statement

The authors declare that the research was conducted in the absence of any commercial or financial relationships that could be construed as a potential conflict of interest.
