# Supplementary material for: Candida spp. Determination and Th1/Th2 Mixed Cytokine Profile in Oral Samples From HIV+ Patients With Chronic Periodontitis
Source: Front Immunol. 2019 Jun 27;10:1465. doi: 10.3389/fimmu.2019.01465 (PMC6610488; doi:10.3389/fimmu.2019.01465)
Supplement: Supplementary file 1 [file Table_1.DOCX]

**Supplementary table 1.** *Candida spp.* isolations distribution in our study groups.

|  | Group A  n (%) | Group B  n (%) | Group C  n (%) | Group D  n (%) | Total  n (%) |
| --- | --- | --- | --- | --- | --- |
| *C. albicans* | 40 (72.7) | 20 (55.5) | 10 (76.9) | 41 (58.6) | 138 (67.7) |
| *C. glabrata* | 9 (16.4) | 4 (11.1) | 2 (15.4) | 10 (14.3) | 48 (23.5) |
| *C. tropicalis* | 0 (0) | 3 (8.3) | 0 (0) | 3 (4.2) | 10 (4.9) |
| *C. krusei* | 0 (0) | 1 (2.8) | 0 (0) | 1 (1.4) | 7 (3.4) |
| *C. dubliniensis* | 0 (0) | 1 (2.8) | 0 (0) | 0 (0) | 1 (0.5) |
| *C. albicans + C. glabrata* | 4 (7.3) | 5 (13.9) | 1 (7.7) | 10 (14.3) | - |
| *C. albicans + C. tropicalis* | 0 (0) | 0 (0) | 0 (0) | 1 (1.4) | - |
| *C. albicans + C. krusei* | 2 (3.6) | 1 (2.8) | 0 (0) | 2 (2.9) | - |
| *C. glabrata + C. tropicalis* | 0 (0) | 0 (0) | 0 (0) | 2 (2.9) | - |
| *C. albicans + C. glabrata + C. tropicalis* | 0 (0) | 1 (2.8) | 0 (0) | 0 (0) | - |
| *Total* | 55 (100) | 36 (100) | 13 (100) | 70 (100) | 204 (100) ^§^ |

The table shows the distribution of the Candida spp. from the 204 strains isolated, as well as the combinations found in our study populations. The total amount and percentage are shown and separated by study groups. As more than one strain could be isolated from a single patient, the amount differs from the total study population.

§ 204 includes the total amount of single, double and triple Candida spp. isolations. Group A: patients with CD4 ≤ 200 cells/μL treated with HAART; Group B: patients with CD4 ≤ 200 cells/μL without HAART; Group C: patients with CD4 > 200 cells/μL without HAART; Group D: patients with CD4 > 200 cells/μL treated with HAART.
